# Supplementary material for: Ferroelectric Dynamic‐Field‐Driven Nucleation and Growth Model for Predictive Materials‐To‐Circuit Co‐Design
Source: Adv Mater. 2026 Jun 13;38(40):e73722. doi: 10.1002/adma.73722 (PMC13378263; doi:10.1002/adma.73722)
Supplement: Supplementary file 1 — Supporting File: adma73722‐sup‐0001‐SuppMat.docx. [file ADMA-38-e73722-s001.docx]

**Supporting Information for**

**Ferroelectric dynamic-field-driven nucleation and growth model for predictive materials-to-circuit co-design**

*Yi Liang*, Soohyeon Kim, Tony Chiang, Megan K. Lenox, Ian Mercer, John J. Plombon, Jon-Paul Maria, Jon F. Ihlefeld, Wenhao Sun, Wei Lu, John T. Heron**

Y. Liang, T. Chiang, J. T. Heron

Department of Materials Science and Engineering, University of Michigan, Ann Arbor, MI 48109, USA

The Ferroelectronics Laboratory, University of Michigan, Ann Arbor, MI 48109, USA

E-mail: [liangyy@umich.edu](mailto:liangyy@umich.edu) [jtheron@umich.edu](mailto:jtheron@umich.edu)

S. Kim, W. Lu

Department of Electrical Engineering and Computer Science, University of Michigan, Ann Arbor, MI 48109, USA

M. K. Lenox

Department of Materials Science and Engineering, University of Virginia, Charlottesville, VA 22904, USA

I. Mercer, J.-P. Maria

Department of Materials Science and Engineering, The Pennsylvania State University, University Park, PA, 16802 USA

J. J. Plombon

Technology Research, Intel Corporation, Hillsboro, OR 97124, USA

J. F. Ihlefeld

Department of Materials Science and Engineering, University of Virginia, Charlottesville, VA 22904, USA

Charles L. Brown Department of Electrical and Computer Engineering, University of Virginia, Charlottesville, VA 22904, USA

W. Sun

Department of Materials Science and Engineering, University of Michigan, Ann Arbor, MI 48109, USA

1. **Deficiency of KAI model in capturing switching behavior under time-dependent voltage**

KAI model describes the transformed fraction *f* of polarization at time *t* by: $\begin{aligned} f\left( t \right)=1-\exp\left[ -\left( \frac{t}{t_{0}} \right)^{n} \right] \#\left( S1 \right) \end{aligned}$

where *t_0_* is the characteristic time related to voltage-independent nucleation rates and domain wall velocities, and *n* is the Avrami exponent related to domain growth dimension. The conventional form of the KAI model only applies to the constant electric field case. To involve voltage dependence, Merz law can be introduced to *t_0_*, i.e. $t_{0}\left( t \right)=t_{\infty}\exp\left[ \frac{V_{a}}{V\left( t \right)} \right]$, so that $\begin{aligned} f\left( t \right)=1-\exp\left[ -\left( \frac{t}{t_{\infty}\exp\left[ \frac{V_{a}}{V\left( t \right)} \right]} \right)^{n} \right] \#\left( S2 \right) \end{aligned}$

$t_{\infty}$ is the characteristic time at infinite voltage, *V_a_* is the activation voltage of domain wall depinning, and $V\left( t \right)$ is the instantaneous voltage across the ferroelectric capacitor. With the voltage-dependent KAI model, the polarization response under the ramp (Figure 1b, green dashed line) coalesces with that under constant bias (Figure 1b, green solid line) once the set point is reached. This behavior is unphysical, as the applied voltage during the ramp remains below the set point prior to 10 ns, and the polarization would therefore be expected to require a longer time to reach the same value. Therefore, a model that captures the voltage path dependence is needed to correctly reflect the polarization switching physics.


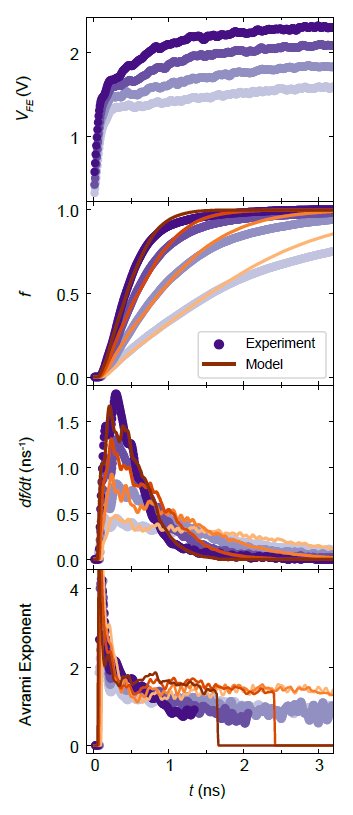


**Figure S1: Fitting HZO transients with both heterogeneous and homogeneous nucleation.** The experimental transients of a 3 $\mu m$ diameter HZO capacitor is fit to the model using Equation (S5) with 5 parameters, including heterogeneous and homogeneous nucleation terms. The model yields parameters $d=0.808\pm0.004$, $V_{a}=11.68\pm0.12 V$, ${\sigma^{'}}^{2}=42.87\pm\infty V$, $A=1918\pm138 ns^{-1}$, and $B=1587\pm\infty ns^{-\frac{1}{d}-1}$. Despite that the modeled data (orange) matches relatively well to the experimental data (purple), the model cannot produce a converged solution to parameter ${\sigma^{'}}^{2}$ and *B*. Moreover, the unreasonably high domain wall energy indicates that homogeneous nucleation is unlikely in the HZO system. Therefore, the polarization switching in HZO is solely dominant by heterogeneous nucleation.

1. **The extraction of material parameters for BTO**

Following the fit procedure in Methods, the experimentally measured switching transients of a 3 $\mu m$ BTO capacitor is fit to the model, as shown in **Figure 2b**. The data best fits the model with a nucleation rate including both heterogeneous and homogenerous nucleation, i.e. summing Equation 3 and 5. Therefore, the total $\left\langle N\left( t \right) \right\rangle$ is given by:

$$\begin{aligned} \left\langle N\left( t \right) \right\rangle=A^{d} \left[ \int_{0}^{t} \exp\left[ -\frac{V_{a}}{V\left( t^{'} \right)} \right] dt^{'} \right]^{d}+B^{d}\int_{0}^{t} \exp\left[ -\frac{V_{a}+{\sigma^{'}}^{2}}{V\left( \tau\right)} \right]\left[ \int_{\tau}^{t} \exp\left[ -\frac{V_{a}}{V\left( t^{'} \right)} \right]dt^{'} \right]^{d}d\tau\#\left( S3 \right) \end{aligned}$$

The extracted parameters are $d=1.144\pm0.004$, $V_{a}=0.536\pm0.003 V$, ${\sigma^{'}}^{2}=1.47\pm0.04 V$, $A=6.13\pm0.07 ns^{-1}$, and $B=713\pm44 ns^{-\frac{1}{d}-1}$. Specifically, the Avrami exponent of BTO shows a distinct behavior compared to HZO, which comes from the contribution of homogeneous nucleation. The universal applicability of the model under dynamic field is manifested by good fits to the switching transients of both polycrystalline materials and single crystalline materials under various distorted waveforms. The reduction of the growth dimension *d* from the physical device dimensions (2D) can be attributed to impediment of the domain wall motion in the system such as the finite size effects, grain boundaries and defects, which produces a truncated time cone^[1]^. The truncated time cone also accounts for the deviation between the model and the experimental data when approaching the end of the switching. Besides a higher growth dimension compared to HZO, BTO also exhibits a much lower Merz barrier (*V_a_*) and heterogeneous nucleation density (*A*). The domain wall energy $\sigma$ of BTO is derived to be ~4.4 mJ/m^2^, according to ${\sigma^{'}}^{2}=\frac{\pi t_{FE}^{2}\sigma^{2}}{2P_{S}k_{B}T}$, and using $P_{S}=20 \mu C/cm^{2}$, $t_{FE}=20 \mathrm{nm}$, $T=300 K$. The domain wall energy derived by our model is comparable to *ab initio* calculated values^[2]^. Homogeneous nucleation density is estimated as ~10^-4^ nm^-d^ using a dipole attachment frequency $\omega_{0}$ of 1 THz^[3]^, and *v_0_* of 5000 m/s^[4]^. It reveals that nucleation in BTO is sparse and that the domains are large (100s-1000s nm).

1. **Material parameters extraction in AlBN**

Due to the limitation of the experimental setup, which can only handle votlages up to 12 V, 10 nm thick AlBN thin film is selected in this study, which only requires a switching voltage of 7-9 V. The experimental data best fit a 3-parameter DFNG model with heterogeneous nucleation. The extracted parameters are $d=0.992\pm0.004$, $V_{a}=66.0\pm0.6 V$, $A=0.71\pm0.06 ns^{-1}$. The experimental data and model fit results are shown in **Figure 2c**.

We note that the leakage current is non-negligible in this ultrathin sample as well as those widely reported in the literature^[5,6]^. The leakage current can be nonlinearly convoluted with voltage and polarization^[7]^, as it cannot be fully accounted for by subtracting the U pulse current in a standard PUND measurement (**Figure S2**). To the best of our knowledge so far, there is no established approach in the community to handle the correlated leakage easily and accurately. Therefore, we use an approximation (**Figure S3**) where the leakage current is forced to saturate to a constant value with a linear ramp from zero, so that the ferroelectric switching current (*I_FE_*) can decay to zero at the end of the switching process. Specifically, we first calculate a current ($I_{P}-I_{U}$) by subtracting the U pulse current from the P pulse current. Then the peak position of the second derivative of this current ($I_{P}-I_{U}$) determines the end of the linear ramp of the leakage current (Figure S3a), because the maximum curvature in the current transient may indicate a transition from the ferroelectric displacive origin to some domain relaxation/ domain wall conduction origin. The static value of the leakage current is taken at a time equal to three times the peak position. With this method, the saturation polarization is consistently saturate to comparable values (155-165 $\mu C\cdot cm^{-2}$) across different supply voltages. The leakage level is much smaller than the ferroelectric switching current ($\leq$10%). As a result, though different subtraction methods may affect the exact model fit parameters, they are unlikely to change the order of magnitude of the physical variables and the projected mechanism.


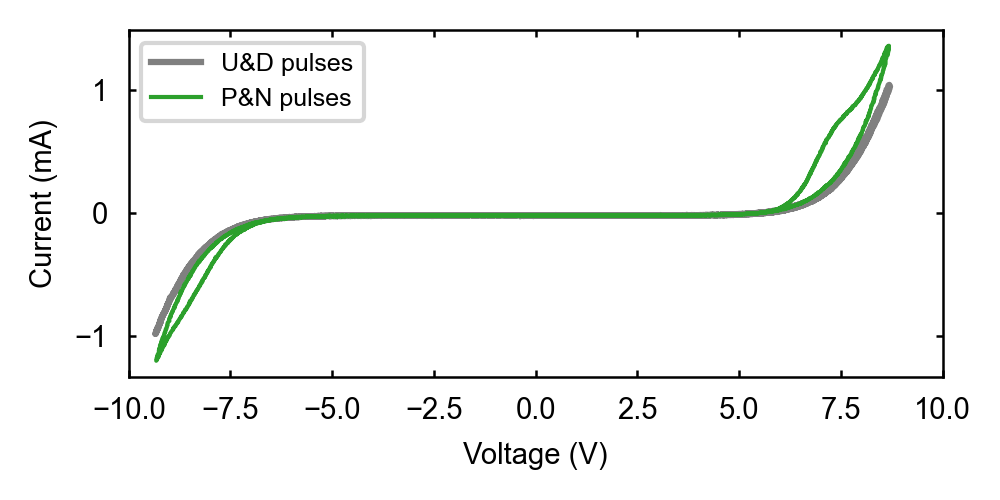


**Figure S2: Leakge current in ultrathin AlBN.** The leakage current is nonlinearly convoluted with voltage and polarization.

**
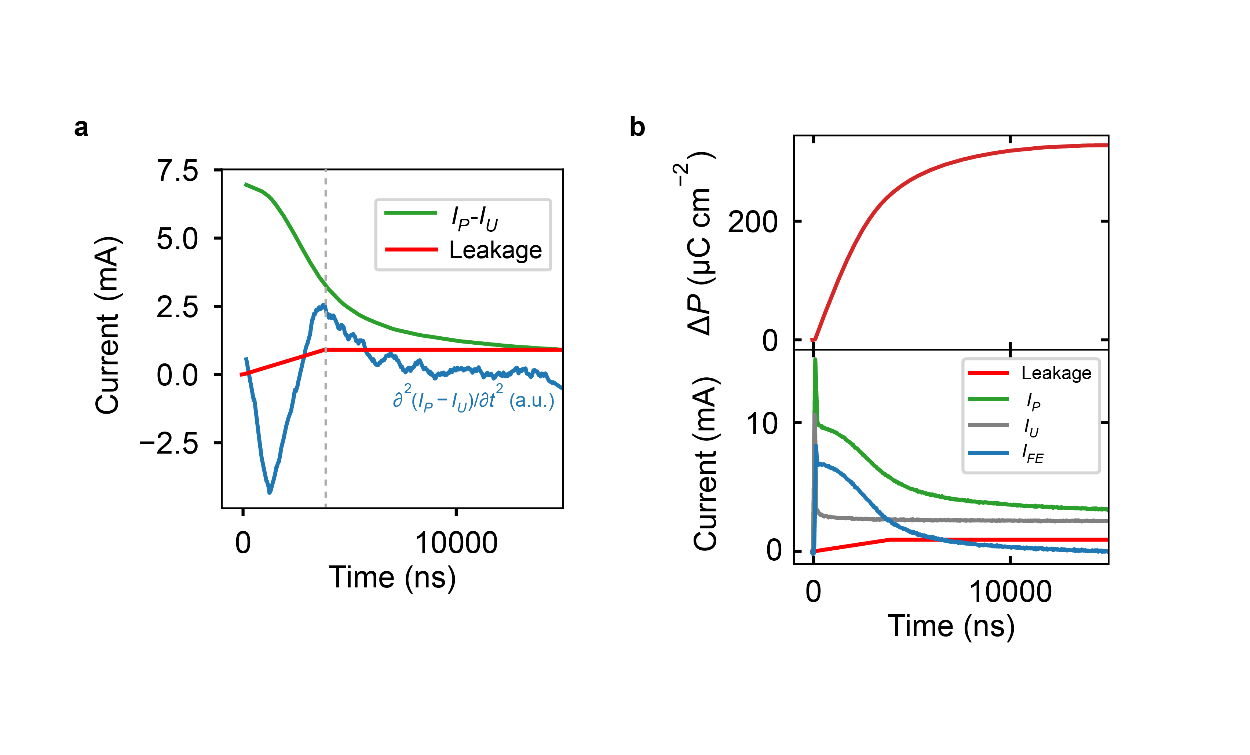
**

**Figure S3: Leakage subtraction. a,** The leakage current is assumed to be a linear ramp with a duration determined by the peak position of the second derivative of ($I_{P}-I_{U}$), and to a static value determined at a time equal to three times the peak position. **b,** With the leakage subtracted, the ferroelectric current can decay to zero and polarization can saturate.

1. **Parameter space exploration and application metrics extraction**
   1. **Simulation of hysteresis loops**

Leakage, linear dielectric constant, and depolarization in the ferroelectric capacitor are ignored and a preset -*P_S_* state is assumed. The method to generate the polarization switching during a voltage ramp is shown in **Figure S4a** and **S4b**, which is essentially half of a hysteresis loop. The voltage ramp is described by $V_{FE}\left( t \right)=V_{0}\cdot\frac{1}{T/4}\cdot t$. *T* is the period of the hysteresis loop, and the frequency *F* of the loop is given by 1/*T*. *V_0_* is the set voltage of the ramp. The frequency of the loops in **Figure 5a** is 1 MHz, and the set voltage is 3 V. The full hysteresis loop is shown in **Figure S4c**. The polarization only switches during 0-250 ns and 500-750 ns per the assumption, and the switching transients in these two periods are asymmetric. Interestingly, this method can be used to generate frequency dependent hysteresis loop. The frequency dependent coercive voltage (*V_C_*) matches the empirical power law $V_{C}\propto F^{\beta}$ ^[8]^(**Figure S5**).

For parameter space exploration, we iterate every combination of parameters among *d* = 0.6, 0.8, 1.0, 1.2, 1.4, 1.6, 1.8; *V_a_* = 4, 8, 13, 18 V; *A* = 10, 10^2^, $5\times{10}^{2}$, 10^3^, $5\times{10}^{3}$, 10^4^, $5\times{10}^{4}$, 10^5^ ns^-1^.

**
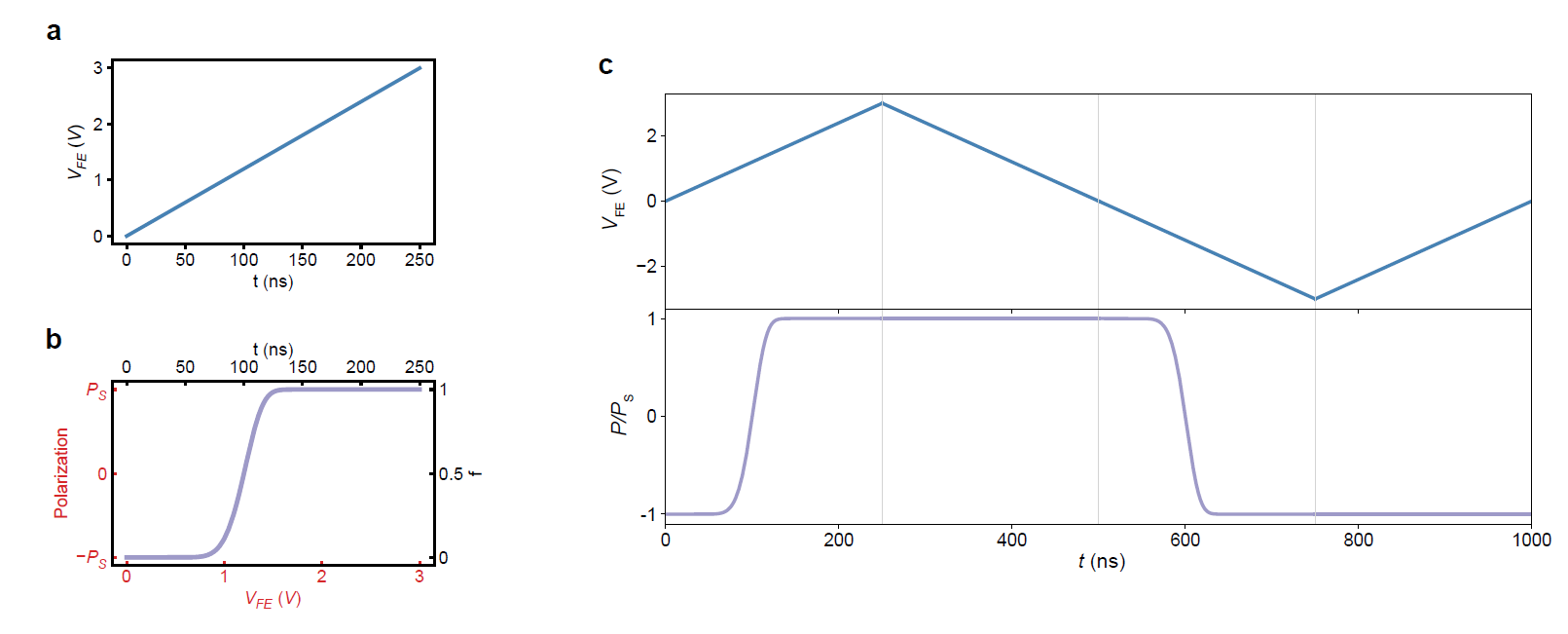
**

**Figure S4: Hysteresis loop generation.** **a,** The voltage ramp of a 1 MHz hysteresis loop. **b,** The transformed fraction *f* as a function of time can be mapped to the polarization as a function of voltage. The ferroelectric polarization is preset to $-P_{S}$, and switches to *P_S_* during the voltage ramp, corresponding to a transformed fraction from 0 to 1. **c,** The full waveform of a hysteresis loop measurement and the corresponding polarization values.


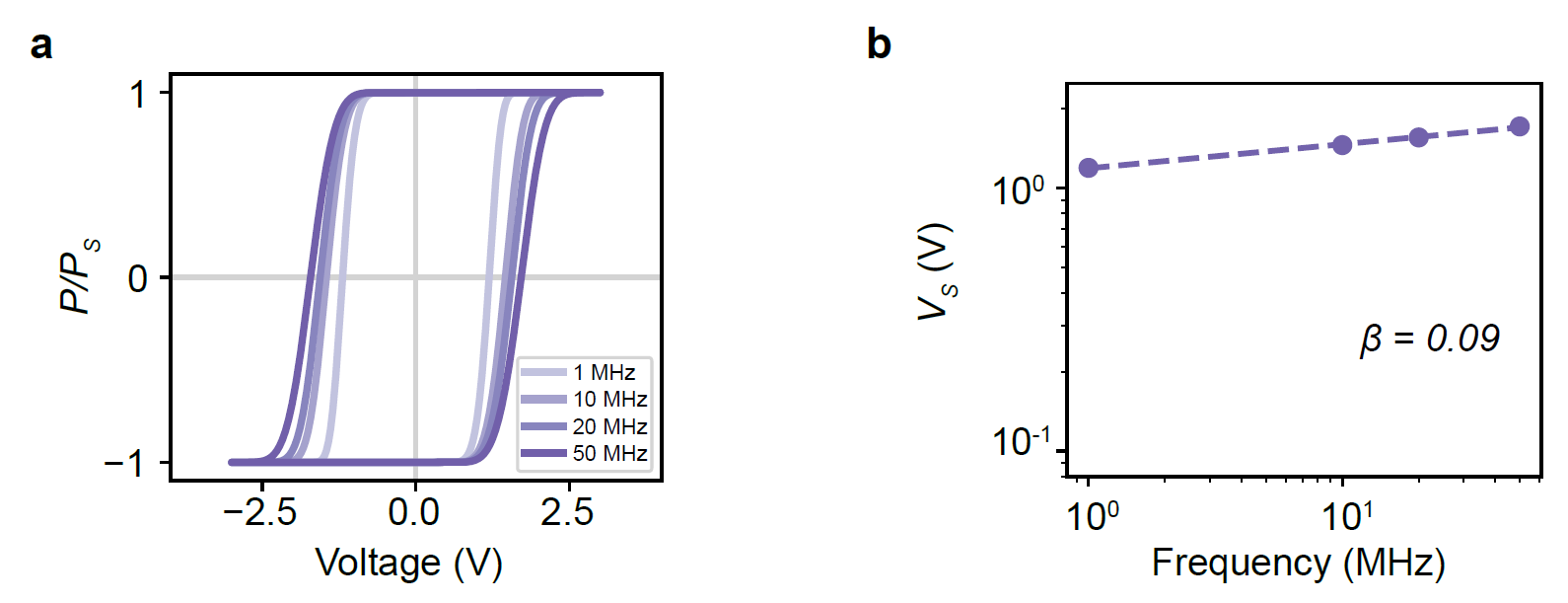


**Figure S5: Frequency dependent hysteresis loops.** **a,** Simulated hysteresis loops under 1, 10, 20, 50 MHz triangular waveform. **b,** Frequency dependent coercive voltage. Using the parameter sets of the 3 µm HZO capacitor, the model captures the empirical power law $V_{C}\propto F^{\beta}$. The dots are the simulated coercive voltages, and the dashed line is a linear fit. The linear fit gives a $\beta$ value of 0.09.

- 1. **Extraction of application metrics and grading of the loops**

Instead of triangular waves and linear ramp of voltages, pulse waveforms are more commonly used in circuits operations for higher latency. All simulated pulse waveforms used in this work are defined as $V_{FE}(t)=\frac{2}{\pi}V_{0}\arctan50t$. *V_0_* is the pulse amplitude. This equation gives a rise time of ~80 ps to reach ~0.85*V_0_*. A simulation example is shown in **Figure S6**.

To grade the loops according to the procedure mentioned in the main text, we first simulate the switching transient under a pulse operation of 1.5 V for every parameter set involved. Then we select the parameter sets that allow more than 99% switching fraction at 10 ns. The voltage when *f* = 0.99 is minimum pulse amplitude required (*V_min_*). We run the simulation again under a pulse with an amplitude of *V_min_* to extract the delay, which is the time needed to complete 99% switching transformation, as well as under a pulse with an amplitude of *V_min_*/2 and a width of the delay to extract $\Delta f$ (normalized disturbed polarization in *V_DD_*/2 write scheme).

For FeCap and FeFET devices, the key metrics are memory windows, namely the capacitance (*C_norm_*) and coercive voltage (*V_C_*), respectively. These two quantities are highly susceptible to the frequency of the electrical waveform, or equivalently the ramp rate of the voltage under a variety of application contexts. As a proof-of-concept, we extract these two values using a fast voltage ramp of 0.1 V/ns, which is approximate to a 320 MHz, 0.05 V AC small signal oscillation.


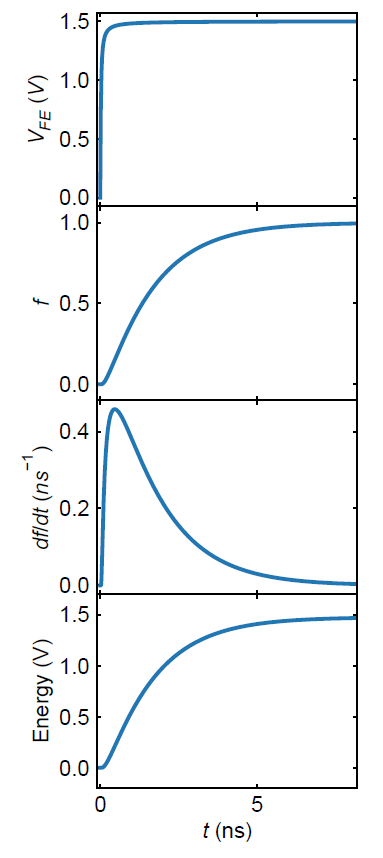


**Figure S6: Simulated switching transient under pulse waveform.** In this simulation, *V_0_* is set to be 1.5 V. After calculating the transformed fraction *f* and then the normalized ferroelectric current *df/dt*, energy is given by integrating the product of *df/dt* and *V_FE_* over time.

1. **Parameter sets filtering for FeFETs and FeCaps**

We use a nominal criterion of *V_C_* ≥ 1.4 V to select favorable FeFETs gate materials. It is inferred from **Figure 5b** that only the delay has a broad distribution and needs consideration under this selection. Table S1 is a table of the satisfying parameter sets, in the order of increasing delay. The parameter set that gives the minimum delay is highlighted in orange. The one that is closest to our measured HZO sample is highlighted in blue. It indicates a possible solution that if the depinning field is relatively unchanged, by slightly increasing the growth dimension (for example through geometric design or removing grain boundaries) and nucleation density (for example by introducing point defects), the HZO can be more suitable for FeFETs. Moreover, the parameter set highlighted in green also provides a potentially viable solution that by diminishing the pinning defects (and therefore increasing *d*, and reducing *V_a_* and *A*), HZO can be adapted to fit the device need.

*C_norm_* ≥ 12 V^-1^ is selected for FeCap devices. From **Figure 5b**, this criterion gives small delay but a wide distribution in other metrics. If $\Delta f$ is required to be less than 1%, only three sets of parameters satisfy the standard (Table S2). These parameter sets have high growth dimension approaching the physical dimension (2D) and small depinning field, which is more aligned with single crystalline materials. However, the nucleation density needs to maintain high. The demanding values and the rarity of suitable parameters indicate polycrystalline materials are probably not ideal for FeCAPs.

| *d* | *V_a_* (V) | *A* (ns^-1^) | Delay (ns) | *V_C_* (V) |
| --- | --- | --- | --- | --- |
| 1.8 | 13 | 5000 | 3.25 | 1.44 |
| 1.6 | 13 | 5000 | 3.56 | 1.44 |
| 1.4 | 13 | 5000 | 4.01 | 1.44 |
| 1.8 | 18 | 100000 | 4.54 | 1.52 |
| 1.2 | 13 | 5000 | 4.73 | 1.43 |
| 1.6 | 18 | 100000 | 4.98 | 1.52 |
| 1.8 | 8 | 100 | 5.24 | 1.45 |
| 1.4 | 18 | 100000 | 5.62 | 1.52 |
| 1.6 | 8 | 100 | 5.79 | 1.45 |
| 1.0 | 13 | 5000 | 5.95 | 1.42 |
| 1.4 | 8 | 100 | 6.58 | 1.44 |
| 1.2 | 18 | 100000 | 6.61 | 1.51 |
| 1.2 | 8 | 100 | 7.83 | 1.43 |
| 1.0 | 18 | 100000 | 8.33 | 1.51 |
| 1.8 | 18 | 50000 | 8.44 | 1.60 |
| 0.8 | 13 | 5000 | 8.48 | 1.41 |
| 1.6 | 18 | 50000 | 9.30 | 1.60 |
| 1.0 | 8 | 100 | 9.98 | 1.42 |

**Table S1: Favorable parameters for FeFETs.**

| *d* | *V_a_* (V) | *A* (ns^-1^) | *C_norm_* (V^-1^) |
| --- | --- | --- | --- |
| 1.6 | 4 | 10000 | 12.51 |
| 1.8 | 4 | 5000 | 12.37 |
| 1.8 | 4 | 10000 | 14.06 |
|  |  |  |  |

**Table S2: Favorable parameters for FeCAPs.**

1. **SPICE modeling implementation**

The SPICE simulation was configured using an equivalent circuit model to capture the transient switching dynamics of the ferroelectric capacitor with interconnect parasitic effects. The setup assumes a HZO ferroelectric capacitor of 3 $\mu m$ in diameter with the material parameters extracted by the DFNG model ($d=0.699$, $V_{a}=12.75 V$, $A=3236 ns^{-1}$), a saturation polarization (*P_S_*) of $15 \mu{C/cm}^{2}$, and an intrinsic linear capacitance of 300 fF. The ferroelectric capacitor is connected to a 3 $\mu m$ wide Cu interconnect with $3 \mu m$ line space. Thus the interconnects produce a parasitic resistance of 0.4 Ω and a line-to-line capacitance of 6.207 fF. A square pulse of 1.5 V with an arctangent-shaped rising profile was employed as the input signal (*V_in_*), $V_{in}=1.5\cdot\frac{2}{\pi}\cdot\arctan10t$.

To implement the DFNG model in the SPICE platform, the equations in the DFNG model need to be represented by physical equivalent circuit components. Specifically, the ferroelectric capacitor is modeled with a linear capacitor *C_DE_* and a switching element modeled as a voltage-controlled current source *I_FE_*, as shown in **Figure S7a**. *I_FE_* is determined by a virtual voltage *V_f_*, which is in turn modeled via a virtual integrator and two voltage-controlled voltage sources, as shown in **Figure S7b**. The integrator sub-circuit consists of a voltage-dependent current source $I_{int}=\exp\left( -\frac{V_{a}}{V_{FE}} \right)$ that feeds into a capacitor to continuously integrate the switching history based on the applied voltage *V_FE_*, yielding an internal state variable *V_out_*. Then, the first voltage-controlled voltage source converts *V_out_* into the variable ${V_{N}=A}^{d}{V_{out}}^{d}$, equivalent to the variable $\left\langle N\left( t \right) \right\rangle$ in the DFNG model. The second voltage-controlled voltage source generates $V_{f}=1-\exp\left( -V_{N} \right)$, which represents the fraction of switched region *f* in the DFNG model. *V_f_* is then used to produce the ferroelectric switching current *I_FE_* in the sub-circuit in Figure S7a. Response from the linear capacitor *C_DE_* and *I_FE_* represents the dynamic FeCAP device, and is fed back to the main equivalent circuit to update the new *V_FE_* values.

The FeCAP cell SPICE model is then used to build circuit simulators in SPICE to evaluate 3$\times$3 and 32$\times$32 FeCap crossbar arrays. To accurately reflect physical routing within the array, interconnect parasitic components in the crossbar arrays are also carefully modeled in SPICE. Specifically, the Pi-network model is used to estimate the distributed resistance and capacitance between adjacent cross-point cells along both the word and bit lines shown in **Figure 6**. A standard *V_DD_*/2 write scheme is implemented to assess the target cell’s switching dynamics and evaluate disturbance on unselected cells. Operating at *V_DD_* = 1.5 V, the selected word line is driven by the abovementioned arctangent-shaped pulse, while the selected bit line is grounded. Simultaneously, all unselected lines are biased at ½ *V_DD_* = 0.75 V to suppress unintended polarization reversal.


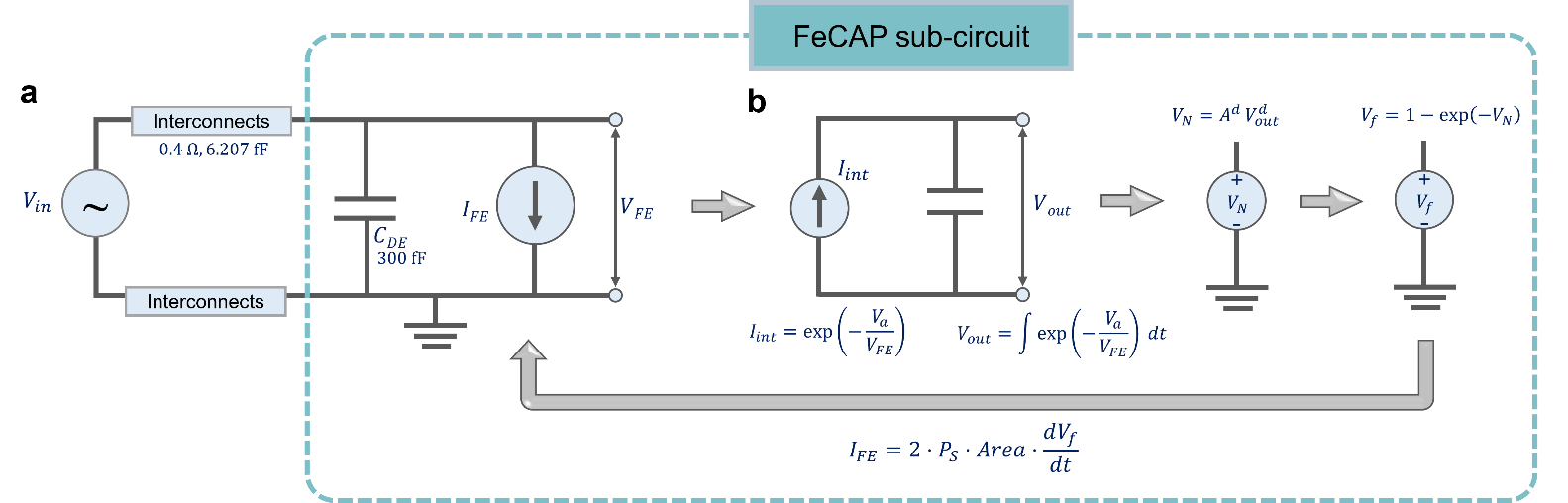


**Figure S7**: **Schematic of the SPICE implementation of the DFNG model to form several sub-circuits that represent a FeCAP cell.** The equations of the DFNG model equations are implemented using corresponding circuit components and sub-circuits as discussed in the text.

1. **Fitting weight analysis**

A weight of 2 has been added to the data points during 0-500 ps for the model fit for HZO and BTO. The primary motivation for introducing weight is to capture the polarization evolution during the rapid voltage variation, which occurs predominantly within the initial ~200 ps. This regime contains significantly fewer data points compared to the full transient, where the time to reach 95% switching ranges from approximately 1.3 ns to 9 ns across the measured cases. To account for ambiguity in defining the rise time of a distorted waveform, this weighted region is extended to 500 ps in the fitting procedure. Without weighting, the fitting algorithm tends to prioritize agreement in the post-500-ps regime.

Here we use the example of HZO to quantify the influence of weighting. The factor applied to the initial 500 ps is systematically varied, and both the total residual sum of squares (RSS) and the RSS in the 0-500 ps window are evaluated (**Figure S8**). As expected, increasing the weight improves agreement within the first 500 ps, but leads to a higher total RSS due to increased deviations towards the end of switching. A weighting factor of 2 is selected as a practical compromise, yielding a closer match in the early-time dynamics while maintaining acceptable overall agreement.

Despite this sensitivity in the fitting procedure, the key physical trends extracted from the model remain robust. Across all weighting conditions examined, the inferred growth dimensionality remains below 1, indicating strongly impeded domain growth. The activation voltage remains significantly higher than the applied voltage and <10% difference for low weights. The derived heterogeneous nucleation density is on the order of 0.2-1 nm^-d^, which is within a reasonable fluctuation range.

Regarding the fit to AlBN, no weight is added to a specific time regime. The rise time (100 ns) is orders of magnitude shorter than the full switching time (10s $\mu$s) and thus the switching dynamic is almost unaffected by the rise time region. However, a weight of 10000 is added to all the normalized current data (*df*/*dt*), as the original values are 4 orders of magnitude smaller than the transformed fraction and the Avrami exponent. The addition of this strong weight is to ensure all the data points can contribute to the fit approximately to the same extent.


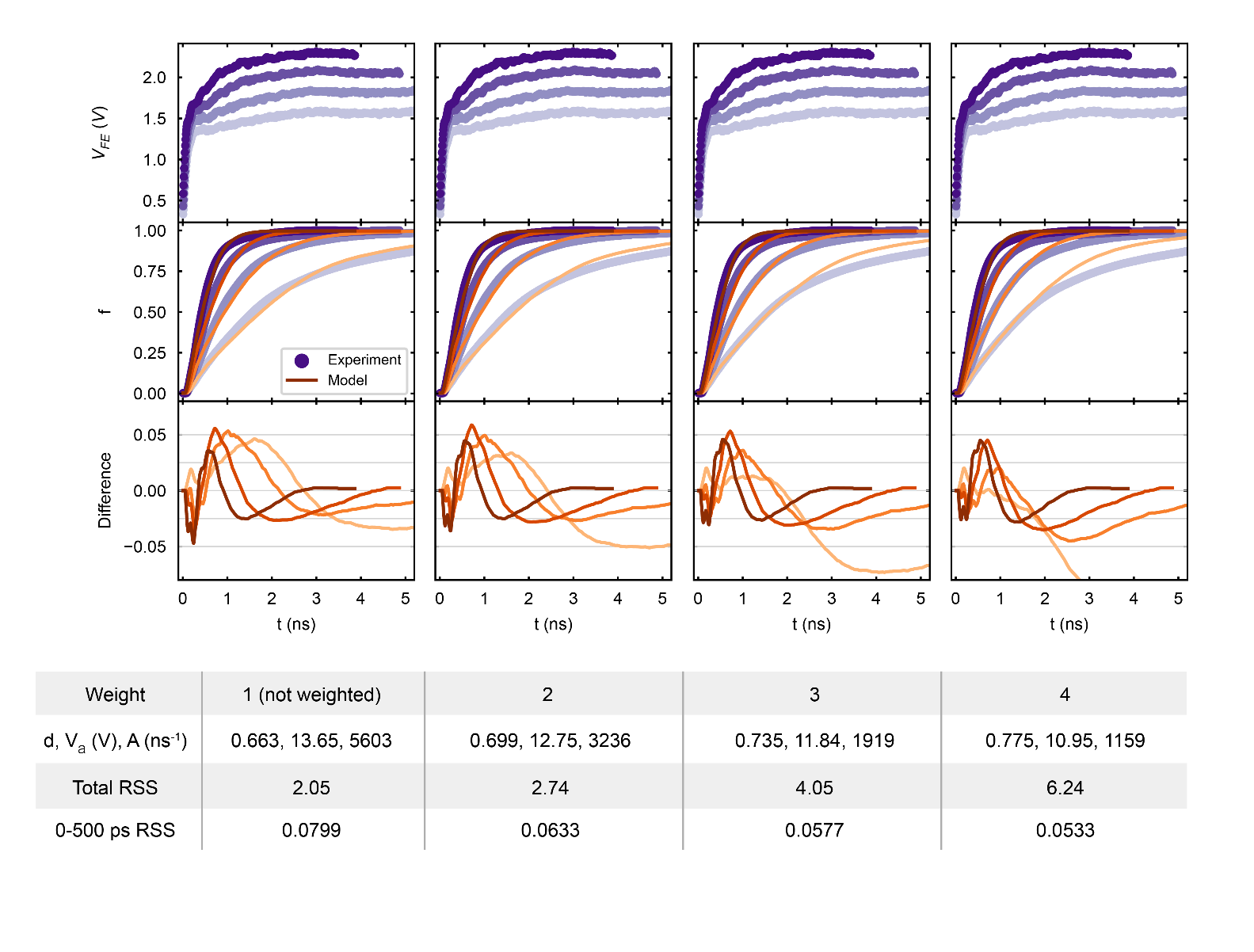


**Figure S8:** **Analysis of fitting method.** The difference between the model-generated and experimentally measured transform fractions are plotted as a function of time, with varying weights from 1 to 4 added to the initial 500-ps stage. The associated fit parameters, total residual sum of squares (RSS) and RSS of the initial 500-ps regime are summarized in the table below.

[1] Concurrent Nucleation and Growth, In *Kinetics of Materials*, John Wiley & Sons, Ltd, **2005**, pp. 533–542.

[2] B. Meyer, *Phys. Rev. B* **2002**, *65*.

[3] J. Hlinka, V. Železný, S. M. Nakhmanson, A. Soukiassian, X. X. Xi, D. G. Schlom, *Phys. Rev. B* **2010**, *82*, 224102.

[4] S. Kashida, I. Hatta, A. Ikushima, Y. Yamada, *J. Phys. Soc. Jpn.* **1973**, *34*, 997.

[5] S. K. Ryoo, K. D. Kim, W. Choi, P. Sriboriboon, S. Heo, H. Seo, Y. H. Jang, J. W. Jeon, M. K. Yeom, S. H. Lee, H. S. Park, Y. Kim, C. S. Hwang, *Advanced Materials* **2025**, *37*, 2413295.

[6] D. Wang, P. Wang, S. Mondal, M. Hu, Y. Wu, T. Ma, Z. Mi, *Advanced Materials* **2023**, *35*, 2210628.

[7] G. A. Salcedo, M. Harrington, S. Nikodemski, V. Vasilyev, M. Newburger, T. Wolfe, C. Schubert Kabban, J. Sattler, A. Islam, *J. Appl. Phys.* **2025**, *138*, 044104.

[8] Z. Chen, Y. Zhang, S. Li, X.-M. Lu, W. Cao, *Appl. Phys. Lett.* **2017**, *110*, 202904.
